# Thermal limits of two biting midges, *Culicoides imicola* Kieffer and *C. bolitinos* Meiswinkel (Diptera: Ceratopogonidae)

**DOI:** 10.1186/1756-3305-7-384

**Published:** 2014-08-20

**Authors:** F Arné Verhoef, Gert J Venter, Christopher W Weldon

**Affiliations:** Flies of Economic Significance Research Group, Department of Zoology and Entomology, University of Pretoria, Private Bag X20, Hatfield, 0028 South Africa; ARC-Onderstepoort Veterinary Institute, Private Bag X5, Onderstepoort, 0110 South Africa

**Keywords:** Bluetongue, African horse sickness, Orbiviruses, Phenotypic plasticity, Thermal biology, Tolerance limits

## Abstract

**Background:**

*Culicoides imicola* Kieffer and *Culicoides bolitinos* Meiswinkel (Diptera: Ceratopogonidae) are both of veterinary importance, being vectors of Schmallenberg, bluetongue and African horse sickness (AHS) viruses. Within South Africa, these *Culicoides* species show a marked difference in their abundances according to altitude, with *C. imicola* highly abundant in lower altitudes, but being replaced as the dominant species by *C. bolitinos* in cooler, high-altitude regions.

**Methods:**

The thermal physiology of field collected adults of each species was determined to evaluate whether it could account for differences in their distribution and abundance. Critical thermal maxima (CT_max_) and minima (CT_min_), as well as upper and lower lethal temperatures (ULT and LLT) were assessed after acclimation temperatures of 19ˌC, 24ˌC and 29ˌC. Critical thermal limits were determined using an ecologically relevant rate of temperature change of 0.06ˌC.min^−1^.

**Results:**

Significant differences in CT_min_ and CT_max_ were found between acclimation temperatures for *C. imicola* and *C. bolitinos.* In *C. bolitinos*, the LLT of individuals acclimated at 24ˌC was significantly improved (LLT_50_ = −6.01ˌC) compared with those acclimated at the other temperatures (LLT_50_ = −4ˌC). Acclimation had a weak (difference in LLT_50_ of only 1ˌC) but significant effect on the LLT of *C. imicola.* When CT_min_, CT_max_, LLT and ULT were superimposed on daily maximum and minimum temperature records from locations where each tested *Culicoides* species is dominant, it was found that temperatures frequently declined below the CT_min_ and LLT of *C. imicola* at the location where *C. bolitinos* was dominant.

**Conclusions:**

The distribution and abundance of *C. imicola* is likely directly constrained by their relatively poor tolerance of lower temperatures. Results for *C. bolitinos* suggest that the adult phase is hardy, and it is hypothesised that the thermal biology of other life stages could determine their range.

**Electronic supplementary material:**

The online version of this article (doi:10.1186/1756-3305-7-384) contains supplementary material, which is available to authorized users.

## Background

Biting midges in the genus *Culicoides* (Diptera: Ceratopogonidae) are incredibly abundant, small flies (ranging from 1–4 mm). Female biting midges feed on vertebrate blood, and in so doing, act as vectors of up to 66 viruses that affect animals including humans [1, 2]. At least three of these viruses, namely bluetongue (BTV), epizootic haemorrhagic disease, and African horse sickness (AHSV) viruses, all belonging within the genus *Orbivirus* (Reoviridae), cause diseases of such international significance in livestock that they have been classified as notifiable to the World Organisation for Animal Health (OIE). The rapid spread of BTV in Europe, outbreaks of AHSV in Spain since 1987 [3], and recent outbreaks of a novel orthobunyavirus, Schmallenberg virus [4, 5], has demonstrated the devastating effect of these viruses on naïve livestock populations. Due to the emergence and over-wintering potential of these viruses in Europe, whereby novel European *Culicoides* species have been incriminated as the vector [6], there is an increased interest in the vector competence and ecology of *Culicoides* species [7]. Similarly, in sub-Saharan Africa where AHS is endemic and widespread, long term economic losses have resulted from export embargoes on horses as well as direct loss of livestock [8]. The population dynamics of *Culicoides* species are markedly influenced by environmental conditions [9]. On account of these factors, there is a need to understand the biotic and abiotic drivers of *Culicoides* distribution and abundance.

Two *Culicoides* species have been implicated in the transmission of BTV and AHSV in southern Africa, namely *C. imicola* and *C. bolitinos. Culicoides (Avaritia) imicola* Kieffer is one of the most widely distributed members of *Culicoides* in the world, extending over the African continent into southern Europe and eastwards to south China [10, 11]. Based on its wide geographical distribution and host preference for larger mammals, as indicated by the high numbers collected near livestock, *C. imicola* is considered the principal vector of AHSV, equine encephalosis virus and BTV in South Africa [12, 13] and BTV in southern Europe [14, 15]. Often found in great densities, with single trap captures able to reach 10^6^ individuals [16], they may make up 99% of all *Culicoides* species collected near livestock in areas where they are abundant [10, 17]. The larvae develop in moist soils rich in organic matter, often in full sun. In years with above-average rainfall, a 200-fold increase in abundance may be observed [16] due to increased availability of breeding sites. In contrast, *C. bolitinos* Meiswinkel occurs in low numbers across sub-Saharan Africa, but can become the dominant species in cooler, high altitude regions [16, 18] such as the Free State province of South Africa, and Lesotho. In South Africa, *Culicoides bolitinos* was also shown to be abundant in the winter rainfall region of the Western Cape province [19, 20], and the dominant *Culicoides* species, in the absence of *C. imicola,* in the sandy dunefields adjoining Port Elizabeth in the Eastern Cape Province [21]. The absence of *C. imicola* at Port Elizabeth in light trap collections made at Struisbaai and Alexander Bay on the southern and western coastline was attributed to the sandiness of the soil [22]. Described in 1989 [11], *C. bolitinos* was identified as a potential vector of AHSV as recently as 1998 [16]. The larval cycle is completed in bovid dung (buffalo, domestic cattle and blue wildebeest) [11]. The abundance of *C. bolitinos* is thus affected by the availability of suitable dung, with the decomposition of dung potentially acting to incubate *C. bolitinos* larvae.

Despite the role of *Culicoides* species as potential vectors and their increased relevance to epidemiological research [23] little is known about their basic physiology. This is most likely due to their small size, nocturnal behaviour as well as ease of injury during handling, which has discouraged or hampered experimental manipulation [10]. As noted above, *C. imicola* and *C. bolitinos* show marked differences in abundance, seemingly according to regional climate. It is likely, therefore, that thermal physiology may be a determining factor in their distribution and abundance. Most of the physiological processes of insects are temperature-dependent [24] and thermal limits are significant for fitness because they determine the ability of an organism to remain active and survive during extreme conditions [25]. Extending from this tenet, thermal limits form a fundamental part of mechanistic niche modelling [26] that aid in the generation of distribution maps and risk management strategies. These distribution models are increasingly important with a changing climate, in order to assist in predicting future outbreaks or range expansions or to assess local changes in dominance of species [27]. Some work has been done on the role of temperature on the geographical distribution of *Culicoides* species [28], including *C. imicola* [29–32], mainly through the use of climate envelope models. The effect of temperature on BTV replication within both *C. imicola* and *C. bolitinos* has also been investigated [33, 34]. However, the tolerance of these species to temperature extremes has never been established, and spatial models are coarse [23]. Knowledge of the thermal biology of these species may aid in understanding their population dynamics, particularly their distribution and abundance, and may even provide insight into the epidemiology of the arboviruses they transmit.

This study determined the thermal limits of adult *C. imicola* and *C. bolitinos*. Phenotypic plasticity in measured thermal tolerance traits was also determined. Results were related to the temperature conditions of locations within the known geographical distribution of these two livestock associated species.

## Methods

### Collections and study animals

Rearing *Culicoides* in the laboratory is extremely difficult [10], and due to unsuccessful rearing attempts during pilot studies, only the thermal limits of adult, field collected individuals were investigated in this study. All midges were collected using Onderstepoort light traps during March, April and October, 2013. Trap catches of both species are highly female biased, so all individuals tested using the methods outlined below were female. All *C. imicola* were trapped at ARC-Onderstepoort Veterinary Institute, Gauteng, South Africa (ARC-OVI; 25ˌ 39’ 2.58” S 28ˌ11’ 8.58” E; 1219 m a.s.l.). The Onderstepoort area is relatively frost-free with only a few days where the minimum temperature will fall below 0ˌC. *Culicoides bolitinos* were trapped on Koeberg farm near the Clarens Valley, Free State, South Africa (28ˌ 0’ 0” S, 28ˌ 31’ 0.12” E, 1631 m a.s.l.). Clarens regularly experiences minimum daily temperatures below freezing, with occasional snow in winter. Both species were transported in cool boxes to the Hatfield Campus of the University of Pretoria where they were housed as described by Venter et al. [35] and fed a 10% sucrose solution (in distilled water) from saturated cotton wool. Adult *C. imicola* and *C. bolitinos* were kept at acclimation temperatures of 19ˌC, 24ˌC and 29ˌC with a 12 L:12D photoperiod for three days before being subjected to thermal tolerance assays. In most cases, insects held at constant temperatures for a period of three days will exhibit phenotypic changes in thermal tolerance traits [36].

Temperature data from meteorological stations close to ARC-OVI (Pretoria-Arcadia, 30164, −25ˌ 44′ 18.8514″ S 28ˌ 12′ 26.3874″ E, 1400 m a.s.l) and Clarens (Golden Gate-Clarens, 30647, −28ˌ 30′ 13.716″ S 28ˌ 35′ 1.68″ E, 1849 m a.s.l) were obtained for the period 1st January 2011 until 31st October 2013 from the ARC-Institute for Soil, Climate and Water (Arcadia, South Africa). These data were used to determine the relevance of the thermal limits of both species.

### Critical thermal limits

A dynamic, ramping temperature protocol was used to determine the critical thermal minimum (CT_min_) and maximum (CT_max_) for each species after having been exposed to the three acclimation temperatures. It has been reported that critical thermal limits vary with methodological context [37], so an ecologically relevant rate of change was determined and used. Little of the micro-habitat preference of adult *C. imicola* is known, except their heliophilic and exophilic nature [31]. Consequently, a known larval habitat and adult catch site was chosen. The appropriate rate of temperature change was determined using iButtons with a 0.5ˌC resolution housed in waterproof silicone enclosures (SL50-ACC06, Maxim, Dallas, TX, USA). The iButtons were buried just beneath the soil surface in two waterlogged areas, in full sun, at ARC-OVI. Emergence traps indicated this area to be a habitat for *C. imicola*. The mean daily temperature change of this microhabitat was found to be 0.06ˌCmin^−1^.

To determine critical thermal limits, individual midges were placed in an aluminium 8-chamber stage, made for use under a dissecting microscope (Additional file 1) and connected to a programmable circulating refrigerated waterbath (CC-K25, Huber Kältemaschinenbau, Offenburg, Germany). A 1:1 blend of propylene glycol and water was pumped through silicone tubing from the waterbath to the stage, through which a number of channels had been drilled to permit flow of liquid and the exchange of heat. Biting midges were placed in individual 0.2 mL plastic microcentrifuge tubes (430–11, WhiteSci, Bellville, South Africa) and subjected to a heating or cooling rate of 0.06ˌC.min^−1^ after a ten minute equilibration period at the start temperature of 24ˌC. Sample size for each treatment was *n* = 14 (two trials, each with n = 7). CT_min_ was defined as the point where muscle contraction ceased (i.e. cold stupor). CT_max_ was initially defined as the onset of muscle spasms [36]. However, no discernible difference between erratic movements due to discomfort or muscle spasms could be seen, and thus CT_max_ was redefined as the heat stupor point, when muscle contractions ceased. In both cases, midges were gently prodded with a horse hair attached to a wooden toothpick to ascertain an inability to move. A type-T thermocouple connected to an eight-channel temperature data logger (TC-08, Pico Technology, St. Neots, UK) was inserted into an empty tube to record temperature in the remaining chamber of the stage.

For each species, data were checked for equality of variances (Levene’s test) and inspected for normality. In all cases, the data met these assumptions. The effect of acclimation on both CT_min_ and CT_max_ were assessed by subjecting data to a one way analysis of variance. Post-hoc multiple comparisons were performed using Fisher’s LSD tests. Analyses were implemented using Statistica v. 7 (Statsoft, Tulsa, Oklahoma, USA).

### Upper and lower lethal temperatures

A static temperature, plunging protocol was adopted to determine lethal temperatures. Ten to 20 individuals from each species and acclimation temperature were placed in 2 ml centrifuge tubes, with 5 replicates per test temperature. The centrifuge tubes were sealed before being placed in a resealable plastic bag that was immersed in a programmable circulating refrigerated waterbath. The fluid (1:1 blend of propylene glycol and water) in the waterbath was held at a set test temperature. The midges were exposed to the test temperature for 2 hours, after which they were placed in a 70 mm Petri dish lined with a moistened 2 cm^2^ filter paper, and given 6 hours to recover. Survival was noted after the recovery period. Temperatures tested were: −6ˌC, −3ˌC and 0ˌC for lower lethal temperatures (LLT), and 36ˌC, 39ˌC and 42ˌC for upper lethal temperatures (ULT). Pilot studies indicated no temperature-related mortality above (for LLT) or below (for ULT) these temperatures and they were omitted. Complete mortality for *C. bolitinos* acclimated at 19 and 29ˌC was recorded at a test temperature of −6ˌC, and so tests at lower temperatures were performed on these flies. *Culicoides bolitinos* acclimated at 24ˌC survived below −6ˌC (100% mortality was recorded at −8ˌC) but this data was not included in analysis of LLT.

Generalised linear models with binomial distribution and logit link function were used to test the effect of test temperature, acclimation and the interaction between test temperature and acclimation on survival. Probit regression analyses were used to determine upper and lower median lethal temperatures (ULT_50_ and LLT_50_). All lethal temperature data analyses were performed in SPSS Statistics 21 (IBM Corporation, Armonk, New York, USA).

## Results

### Critical thermal limits

**Figure 1:**
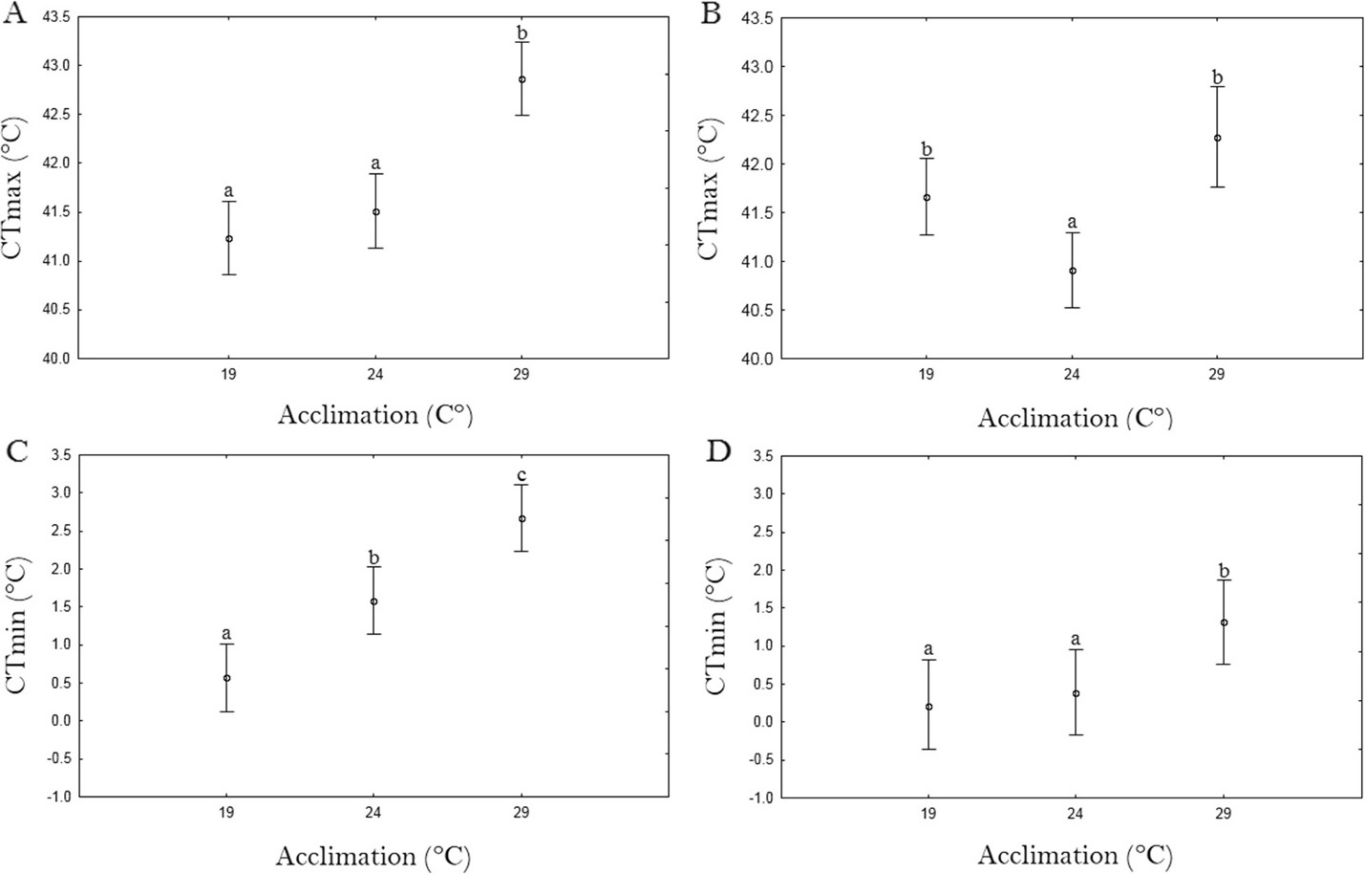
**Effect of acclimation on critical thermal maximum for (A)** ***C. imicola*** **and (B)** ***C. bolitinos*** **, and critical thermal minimum of (C)** ***C. imicola*** **and (D)** ***C. bolitinos*** **.** In each case, the mean ± 1 S.E. is shown (n = 14). Means labelled with the same lowercase letter are not significantly different from each other at the 0.05 level (Fisher’s LSD).

Critical thermal limits for *C. imicola* responded significantly to acclimation (CT_min_: F_2,39_ = 23.553; p < 0.0001; CT_max_: F_2,39_ = 22.2; p < 0.0001) with an increase of 2.10ˌC in CT_min_ and 1.63ˌC for CT_max_ between acclimation temperatures of 19 and 29ˌC (Figure 1). Post hoc comparisons indicated that the CT_min_ of *C. imicola* from each acclimation temperature was significantly different from all other acclimation temperatures. For CT_max_, *C. imicola* acclimated at 19ˌC and 24ˌC did not differ significantly from each other but both differed significantly from the individuals acclimated at 29ˌC.

For *C. bolitinos*, variation in critical thermal limits in response to acclimation was less pronounced than in *C. imicola* (Figure 1; CT_min_: 1.37ˌC; CT_max_: 1.30ˌC), but these differences were statistically significant (CT_min_: F_2,38_ = 4.64907; p = 0.0156; CT_max_: F_2,39_ = 11.3; p = 0.0001). Fisher’s LSD tests revealed that the CT_min_ between 19ˌC- and 24ˌC-acclimated individuals did not differ significantly, but both differed significantly from *C. bolitinos* acclimated at 29ˌC. For CT_max_, the 24ˌC treatment was lowest and differed significantly from the 19ˌC- and 29ˌC-acclimated individuals, which did not differ significantly from each other.

### Lethal temperatures

**Figure 2:**
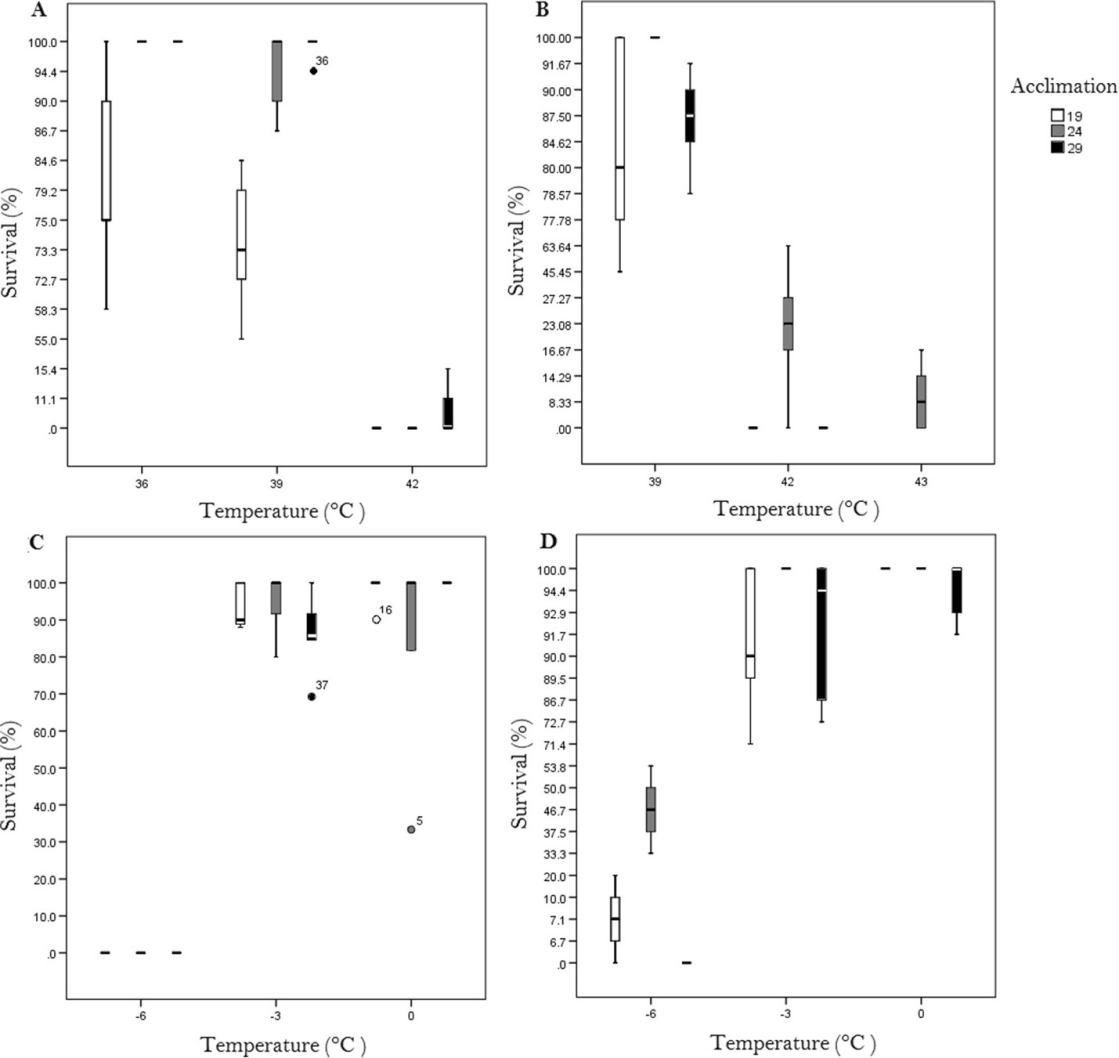
**Effect of test and acclimation temperature on survival of cohorts of** ***C. imicola*** **and** ***C. bolitinos*** **.** Survival was assessed 6 hours after each cohort was exposed to the test temperature for 2 hours. **(A)** Survival at high temperatures by *C. imicola*; **(B)** survival at high temperatures by *C. bolitinos*; **(C)** survival at low temperatures by *C. imicola*; **(D)** survival at low temperatures by *C. bolitinos*. In each case, the mean ± 1 S.E. are shown (n = 5) for each test and acclimation temperature combination.

The interaction of LLT test temperatures and acclimation temperature had a significant effect on survival of both species (Table 1, Figure 2 A and B). *Culicoides bolitinos* acclimated to 24ˌC had the lowest LLT_50_ (−6.01ˌC), whereas those acclimated at 29ˌC had the highest LLT_50_ (−3.98ˌC). Parameter estimates relative to an acclimation temperature of 29ˌC were inspected and indicated that survival of *C. bolitinos* was significantly higher (p < 0.001) when acclimated at 24ˌC. LLT of those acclimated at 19ˌC and 29ˌC were not significantly different from each other. There was no significant effect of acclimation on the LLT of *C. imicola*. The LLT_50_ between acclimation treatments varied by 1ˌC, with the lowest estimated for 19ˌC (−4.39ˌC).

**Table 1:** **Effects of temperature and acclimation on the thermal limits of** ***Culicoides bolitinos*** **and** ***C. imicola***

Species	Trait	Effect	Wald *χ* ^2^	d.f.	p
*C. bolitinos*	ULT	Intercept	276.072	1	<0.0001
		Acclimation	341.425	2	<0.0001
		Temperature	5.826	1	0.016
		Acclimation × Temperature	521.357	5	<0.0001
	LLT	Intercept	59.813	1	<0.0001
		Acclimation	37.235	2	<0.0001
		Temperature	128.514	2	<0.0001
		Acclimation × Temperature	107.761	4	<0.0001
*C. imicola*	ULT	Intercept	4.1	1	0.043
		Acclimation	29.793	2	<0.0001
		Temperature	63.892	2	<0.0001
		Acclimation × Temperature	67.629	4	<0.0001
	LLT	Intercept	167.244	1	<0.0001
		Acclimation	6.978	2	0.031
		Temperature	0.993	1	0.319
		Acclimation × Temperature	425.118	4	<0.0001

Interactions between acclimation and temperature were significant for the ULT of both species (Table 1) Upper lethal temperatures were generally similar for both species, (mean ULT_50_: *C. imicola* = 40.05ˌC; *C. bolitinos* = 40.39ˌC). No significant difference relative to 29ˌC was noted for 19ˌC-acclimated *C. bolitinos,* though individuals acclimated to 24ˌC did differ significantly from 29ˌC, with 24ˌC-acclimated *C. bolitinos* having the highest ULT_50_ (41.59ˌC)*.* For *C. imicola*, parameter estimates relative to 29ˌC indicated a significant difference between the 19ˌC and 29ˌC acclimated flies but none between 24ˌC and 29ˌC. *Culicoides imicola* showed near-total mortality when exposed to 42ˌC, with only 5.3% of the 29ˌC treatment surviving, and 0% of the other treatments. *Culicoides bolitinos* showed similar mortality at 42ˌC, with 0% survival of 19ˌC and 29ˌC. The 24ˌC-acclimated *C. bolitinos,* however, had a survivorship of 26.13%, with complete mortality documented only when subjected to 44ˌC (Figure 2).

## Discussion

Temperature is a determining factor in insect life history, and thermal tolerance plays a key role in determining insect distribution [24, 38, 39]. Critical thermal limits are valuable for determining the relative temperature tolerances of insects acclimated to different temperatures, or originating from different latitudinal and altitudinal ranges [40]. Typically critical thermal minima are more variable in response to acclimation than critical thermal maxima, both inter- and intra-specifically [41, 42]. Interspecific differences in our study agree with this notion, with the CT_max_ of *C. imicola* and *C. bolitinos* being similar (41.87ˌC SE ± 0.976, and 41.56ˌC SE ± 0.828 respectively), whilst the CT_min_ of *C. imicola* (1.61ˌC SE ± 1.175) is nearly a degree higher than that of *C. bolitinos* (0.65ˌC SE ± 1.117). For the *Culicoides* species tested in this study, expected intra-specific variation was found only for *C. imicola*, with acclimation at temperatures from 19 to 29ˌC leading to a change in CT_min_ of 2.10ˌC, but only 1.63ˌC for CT_max_. In the case of *C. bolitinos,* while acclimation had a statistically significant effect on both CT_min_ and CT_max_, the change in CT_min_ (1.37ˌC) was similar to that of CT_max_ (1.30ˌC). For *C. imicola*, CT_min_ changed in a stepwise manner, with approximately 1ˌC difference between acclimation temperatures. *Culicoides bolitinos* showed little variation between the two lower acclimation temperatures (<0.5ˌC), but the 29ˌC-acclimated midges showed a significant increase in CT_min_, possibly because 24ˌC still falls within the optimal performance breadth for *C. bolitinos*, whereas 29ˌC might be considered an ‘extreme’ temperature that reduces performance (see Deere & Chown [43]).

Little difference in the upper lethal temperatures was found between *C. imicola* and *C. bolitinos*, highlighting limited variability in ULT. Similar to critical temperatures, it has previously been found that variability in lethal limits is greater at a family or generic level, with limited variability between species [44]. Upper thermal limits are also less pliable than lower limits, among species, populations, and over time (i.e. plasticity responses) [26, 42] Our study corroborates this, with little difference in survival of *C. imicola* and *C. bolitinos* at high temperatures.. However, our study also points to a dissimilar response of different species to experimental conditions, and possibly environmental variation, as suggested by Chown et al. [37]. *Culicoides imicola* had a clear response to acclimation, with prior exposure to high temperatures improving heat tolerance in a stepwise manner. This was not the case with *C. bolitinos* however, as individuals acclimated to 24ˌC outperformed both those acclimated at 19ˌC and 29ˌC. The relatively poor performance of *C. bolitinos* when exposed to high temperatures after acclimation at 29ˌC contrasts to the idea of the beneficial acclimation hypothesis, and may indicate a deleterious acclimation response. The deleterious acclimation hypothesis predicts substantial negative performance implications for individuals held at extreme temperatures, both relative to those at median temperatures and those that typically do not experience such extremes in the field [43]. Indeed, although not formally tested, *C. bolitinos* acclimated at 29ˌC exhibited higher mortality in holding cages, with no individuals surviving past a week in the laboratory, whereas conspecifics acclimated at 19ˌC and 24ˌC were capable of surviving in the laboratory for at least two weeks. Determining the degree to which stress proteins and other thermal protectants are up-regulated would provide clarification as to whether a temperature of 29ˌC is indeed detrimental [40], as would longevity and other performance assays.

Acclimation had no significant effect on the LLT of *C. imicola,* with LLT_50_ values similar for all acclimation temperatures (*c*. -4ˌC), and total mortality at −6ˌC. In contrast, acclimation had a significant effect on *C. bolitinos*, with the LLT_50_ for the 24ˌC-acclimated individuals reaching −6.01ˌC, but no significant difference between 19ˌC and 29ˌC was found. The deleterious acclimation hypothesis might once again explain the observed pattern, as would a mismatch between optimum temperatures and realised habitat. Another possibility may be the uncontrolled age and physiological state of the field-collected insects. To obtain sufficient numbers for all experiments it was necessary to collect additional individuals from the field by trapping on several occasions. Senescence has been suggested to reduce stress tolerance in insects [41]. This is supported by Luckinbill [44], who found longevity selection improved low temperature tolerance in *Drosophila melanogaster* [41]. Adult *C. bolitinos*, however, have an average life expectancy of approximately 20 days, but may survive up to 90 days [15]. Similarly, *C. imicola* adults can survive for more than 15 days and *C. pycnostictus* for 54 days at temperatures of −1.5ˌC [17]. Given that our collection methods were consistent and that the period of time that elapsed before testing was an equivalent proportion of the life expectancy of both species, we believe that our data are a true representation of the ability of *C. imicola* and *C. bolitinos* to withstand temperature extremes.

**Figure 3:**
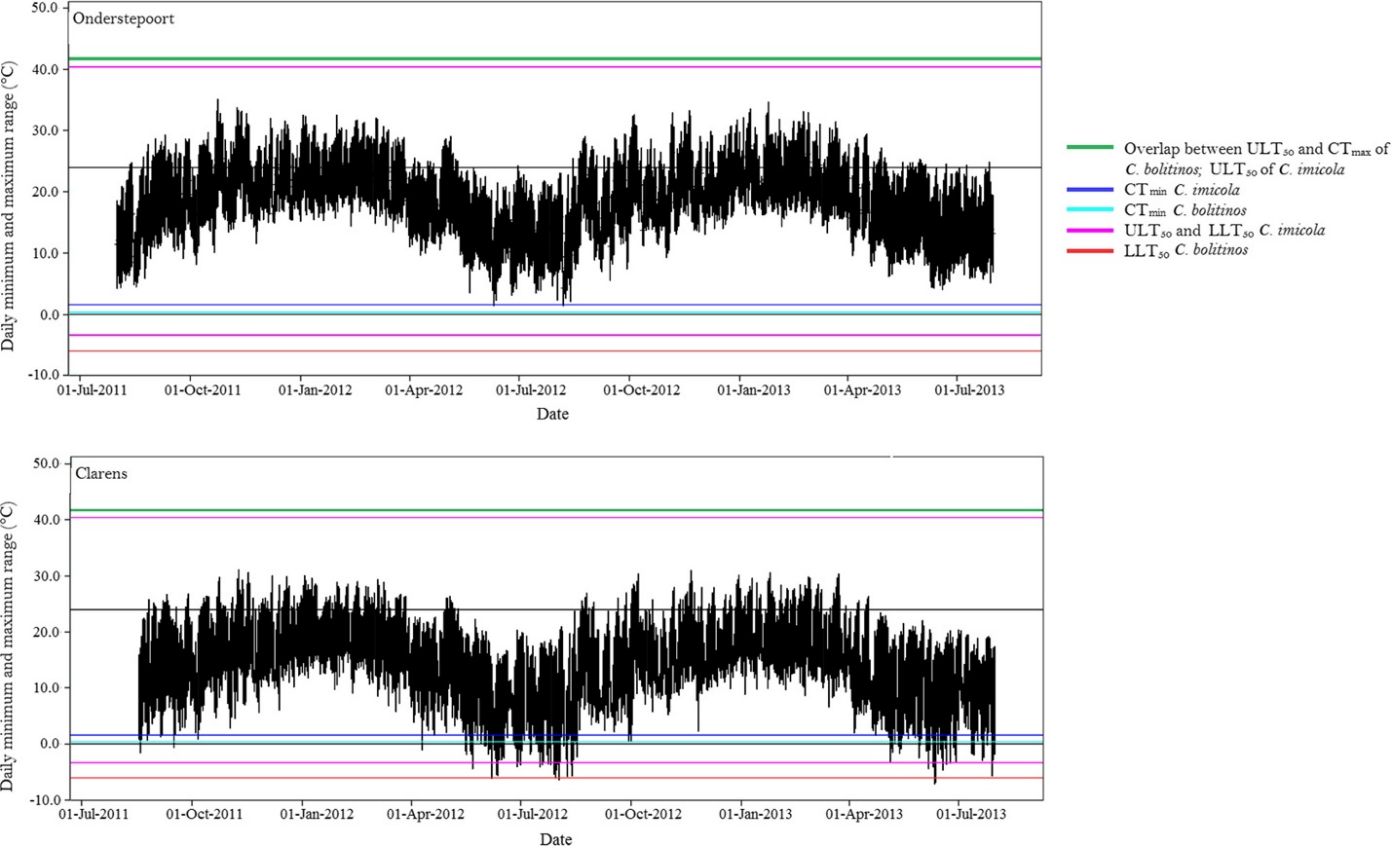
**Daily temperature range for Onderstepoort and Clarens from the end of winter 2011, to end of winter 2013 (1 August 2011 – 31 July 2013).** Horizontal lines represent thermal limits of *C. imicola* and *C. bolitinos*: ULT_50_ and CT_max_ of *C. bolitinos* and ULT_50_ of *C. imicola* (red); ULT_50_ of *C. imicola* (orange); CT_min_ of *C. imicola* (purple); LLT_50_ of *C. imicola* (magenta); CT_min_ of *C. bolitinos* (blue); LLT_50_ of *C. bolitinos* (light blue).

This study indicates that lower temperature limits may play a role in constraining the distribution of *C. imicola*. Modelling attempts often rely on a mean temperature threshold of 12.5ˌC to predict presence or absence of *C. imicola* [29, 45] as established by Purse et al. [46] *.* Peters et al. [45] on the other hand, found absence indicated by a mean temperature of 14.4ˌC and presence by a mean of 15.7ˌC. For the two years of weather data we analysed, Clarens had a mean annual temperature of 14.51ˌC, two degrees above the suggested thermal intercept suggested by Purse, but very close to that of Peters and colleagues. Furthermore, the suggested mean minimum temperature thresholds of Peters et al. (Absence: 9.4ˌC; Presence: 10.0ˌC) coincide strongly with the mean minimum temperatures of our study sites (Clarens: 8.67ˌC; Onderstepoort: 10.74ˌC). However, we propose that mean temperatures are only an indication of potential distribution, and it is extreme minimum temperatures reached that determine population persistence of *C. imicola.* The lowest temperature recorded for Clarens in 2012 was −8ˌC (Figure 3), with temperatures regularly below 0ˌC and often dropping below the LLT of adult *C. imicola* in winter. In tandem with a relatively high CT_min_, it seems likely that the thermal environment in the areas surrounding Clarens may prevent adult *C. imicola* from surviving or establishing in any great abundance, concurring with the negative correlation found between *C. imicola* abundance and low winter temperatures [47]. In addition, it is highly likely that the low winter temperatures of Clarens and similar areas affect larval development and survival because the lower developmental threshold of larvae is ~11.75ˌC (calculated from [10]). Indeed, Baylis et al. [30] suggested that minimum temperatures determine the ability of the *C. imicola* to successfully overwinter as larvae (for one or two months [47]), as the larvae inhabit the O horizon and are easily affected by ambient temperature in the open habitats they prefer [31]. Furthermore, Legg et al. [48] suggested that median lower developmental thresholds are useful for determining phenology (and thus also distribution potential).

In the case of *C. bolitinos* it appears that the thermal limits of adults are not related to their distribution because their upper temperature tolerance is similar to that of *C. imicola* and they are tolerant of lower temperatures. It may be that the thermal limits of other life stages, especially the larvae, play an important role in limiting the abundance and distribution of *C. bolitinos*. In warmer environments where they are less abundant, the exothermic decomposition of dung could lead to dung temperatures too high for larvae to survive. Evidence for this scenario is currently not available, as when we placed iButtons in fresh dung, mean dung temperatures were isothermic with moderate ambient temperature (*c.* 14ˌC), making inference difficult. Non-lethal thermal stress effects could also explain their limited numbers in warmer regions. *Scatophaga stercoraria,* another dipteran with coprophagous larvae, displays temperature-mediated variation in egg size as a maternal physiological response [49]. In this study, Blanckenhorn [49] found reduced egg-size and reduced survivorship of offspring even at intermediate temperatures of 24ˌC. Similar stress effects could suppress populations of *C. bolitinos* in hotter environments. Modelling done on an allopatric, identical sister species *C. brevitarsis* in Australia found that mean maximum temperatures could predict BTV infection (and thus *C. brevitarsis* distribution) with an accuracy of ~75% [50]. Considering the notable similarity of *C. bolitinos* and *C. brevitarsis* in morphology, habitat and habit, it is possible that it could be similarly employed.

## Conclusion

It appears that although factors such as precipitation and availability of breeding sites may affect *C. imicola* distribution and abundance, thermal limits play an important role in determining abundance and distribution of this species. Temperatures lower than the CT_min_ of *C. imicola* may prohibit its dominance in high-altitude areas or its expansion into more temperate climes. The presence of *C. imicola* in Clarens, even though at low abundance (<1% of all *Culicoides* spp. captured) [16], indicates that some individuals of this species may utilise protected microclimates to escape severe cold, similar to some European species that overwinter in stables [51]. Another possibility may be that *C. imicola* reinvades these areas annually, as *Culicoides* species have been known to travel long distances by wind-mediated dispersal [52]. The results for *C. bolitinos* are more complex, as adults of this species show a relative wide thermal performance breadth in comparison to *C. imicola*. Further research on the thermal biology of juvenile life stages is required to explain the more temperate distribution of *C. bolitinos.* Due to difficulty in rearing C*ulicoides* in the laboratory, this resolution may prove to be logistically challenging. This study shows that thermal limits and tolerance varies between closely related species and that this may play a regulatory role in the presence and abundance of species. A better understanding of thermal phenomena may contribute to the creation of more realistic risk maps and the elucidation of the influence of climate change on the presence and expansion of viral diseases.

## Electronic supplementary material

Additional file 1: **Technical drawing of water-jacketed eight-chamber stage, made for use under microscope. Each chamber can accommodate a 0.2 ml micro-centrifuge tube.** (TIFF 145 KB)

## Abbreviations

AHSV: African horse sickness virus
BTV: Blue tongue virus
CT_min_: Critical thermal minimum
CT_max_: Critical thermal maximum
LLT: Lower lethal temperature
ULT: Upper lethal temperature.
