# Supplementary figures and images for: Thermal limits of two biting midges, Culicoides imicola Kieffer and C. bolitinos Meiswinkel (Diptera: Ceratopogonidae)
Source: Parasit Vectors. 2014 Aug 20;7:384. doi: 10.1186/1756-3305-7-384 (PMC4150952; doi:10.1186/1756-3305-7-384)

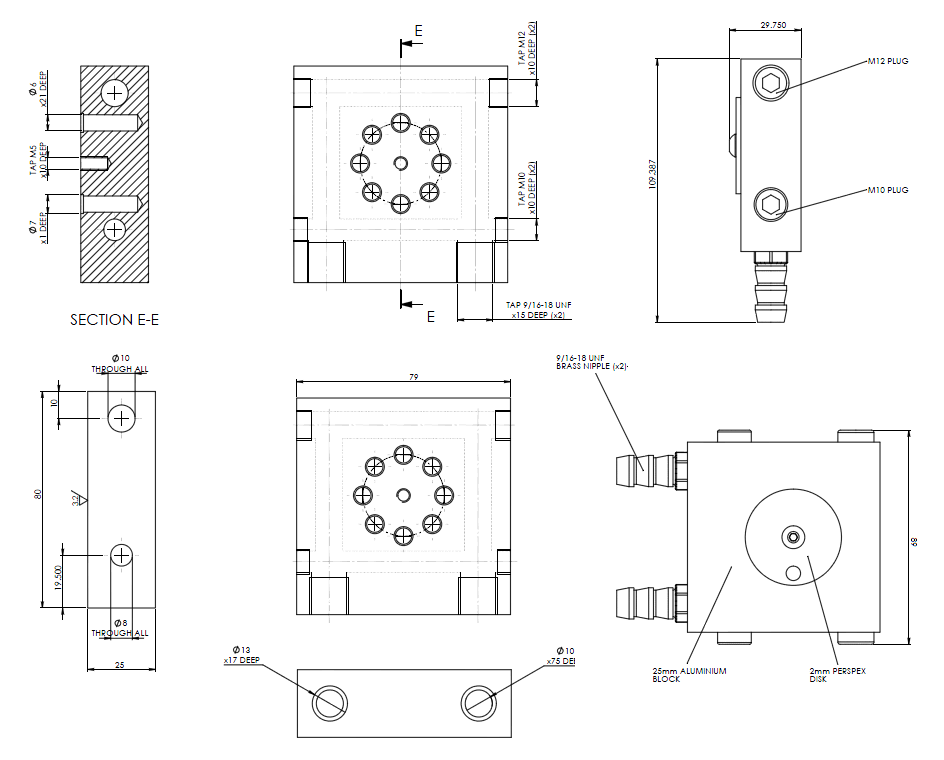

Supplement: Supplementary file 1 — Additional file 1: Technical drawing of water-jacketed eight-chamber stage, made for use under microscope. Each chamber can accommodate a 0.2 ml micro-centrifuge tube. (TIFF 145 KB) [file 13071_2014_1559_MOESM1_ESM.tiff]
